# Nutshells as Efficient Biosorbents to Remove Cadmium, Lead, and Mercury from Contaminated Solutions

**DOI:** 10.3390/ijerph18041580

**Published:** 2021-02-07

**Authors:** Mariana Dias, João Pinto, Bruno Henriques, Paula Figueira, Elaine Fabre, Daniela Tavares, Carlos Vale, Eduarda Pereira

**Affiliations:** 1 Department of Chemistry, University of Aveiro, 3810-193 Aveiro, Portugal; marianadias@ua.pt (M.D.); elainefabre@ua.pt (E.F.); danielatavares@ua.pt (D.T.); 2 LAQV-REQUIMTE & Department of Chemistry, University of Aveiro, 3810-193 Aveiro, Portugal; joao.pedro.pinto@ua.pt (J.P.); paulafigueira@ua.pt (P.F.); eduper@ua.pt (E.P.); 3 CIIMAR–Interdisciplinary Centre of Marine and Environmental Research, 4450-208 Matosinhos, Portugal; carlos.vale@ciimar.up.pt

**Keywords:** water remediation, food wastes, nutshells, heavy metals, contamination

## Abstract

The release of potentially toxic elements into the environment, and their effects on aquatic ecosystems still present a real threat. To avoid such contamination, the use of biological sorbents as an alternative to conventional and expensive water remediation techniques has been proposed. The present study evaluated the potential of 0.5 g L^−1^ of peanut, hazelnut, pistachio, walnut, and almond shells to remove the requisite concentrations of cadmium (Cd), lead (Pb), and mercury (Hg) from contaminated water. Hazelnut shells were identified as the sorbent with the highest potential and were evaluated in mono- and multi-contaminated mineral water. The influence of sorbent-intrinsic and solution-intrinsic characteristics were assessed. Differences among sorbents were attributed to varying percentages of their main components: cellulose, hemicellulose, and lignin. Matrix complexity increase caused a decrease in Cd removal, presumably due to the diminution in electrostatic interaction, and complexation with anions such as Cl^−^. When simultaneously present in the solution, contaminants competed, with Pb showing higher affinity to the sorbent than Hg. High efficiencies (>90%) obtained for hazelnut shells for all elements in ultrapure water and for Pb and Hg in mineral water) reveals the high potential of this low-cost and abundant waste for use in the remediation of contaminated waters (circular economy).

## 1. Introduction

Despite extensive efforts to reduce contamination of the environment, potentially toxic elements (PTEs) such as metals and metalloids are still present in several technological processes, which can lead to the presence of such contaminants in industrial wastes, often released into rivers or into the ocean [1,2]. Untreated, or ineffectively treated wastes from industrial units represent a risk to aquatic systems [3,4,5] since PTEs are persistent, they can be bioaccumulated in organisms, biomagnified along the food chain, and eventually reach humans, posing multiple risks to health [6,7,8]. The United States Environmental Protection Agency (EPA) and the World Health Organization (WHO) have set a maximum limit for all PTE concentrations in various systems [9], which have been translated into legal regulations by countries, like Portugal [10,11]. Furthermore, the Agency for Toxic Substances and Disease Registry (ATSDR) periodically publishes a list of priority hazardous substances that consider the reactivity and toxicity of the element, the amount in the environment, the form of contamination, and the potential human exposure [12]. In this list, arsenic (As), lead (Pb), mercury (Hg), and cadmium (Cd) are included, occupying the foremost positions of the most hazardous substances, which need to be reduced and/or eliminated from the environment.

The attribution of a priority status to mercury, cadmium, and lead (commonly known as the toxic-trio) has promoted the study and development of various novel techniques for their removal from contaminated water, as alternative to classical treatments of coagulation and flocculation, ionic exchange, chemical precipitation, and membrane filtration. Classical treatments have important disadvantages that it is imperative to overcome, namely high operational costs, incomplete removal accompanied by reduced selectivity, and the generation of toxic byproducts such as muds with high concentrations of PTEs [5,13]. In recent decades, the application of biotechnology in reducing the impacts of contamination by PTEs has been gradually becoming an alternative method to conventional treatments [14,15]. One of the developing methods that has attracted attention is biosorption, which is based on the binding of PTEs to various biological materials [16,17]. PTE ions bind to biomass by a complex process comprising various mechanisms. Examples are surface and pore adsorption, ion exchange, microprecipitation, chemisorption (including complexation and chelation), physisorption and entrapment in capillaries and spaces of the polysaccharide network, due to concentration, causing diffusion through the cell wall and membrane [6,13,16,18,19]. These mechanisms differ according to the substance to be sorbed, the nature of the biomass, and its processing [20,21]. An example of an effective sorbent is activated carbon, with a porous structure and high surface area. However, due to its high cost and inability to be regenerated, other sorbents have been explored [22].

In the wide range of biomass used as a biosorbent [23], agricultural residues resulting from the processing or consumption of fruits or vegetables constitute a promising alternative [24,25]. The use of these porous structure materials reflects the paradigm of treating waste with waste [22,26]. Among biosorbents, nutshells have several advantages over other fruit barks and peels, since they are not perishable, have a high content of polysaccharides, great porosity, and no commercial value [2]. Besides that, nutshells are widely available. In the last ten years, the production of nuts has grown at an increasing pace, following the trend of their consumption associated with health benefits [27], reaching about 4.6 million metric tons in season 2019/2020 [27]. Almond and walnut are the most produced, corresponding to more than half of world total share, followed by cashews (17%), pistachios (14%) and hazelnuts (12%) [27]. During nut processing, a considerable amount of by-products are generated, where the shells represent the largest slice (67% of the total weight of the nuts) [28]. These characteristics have sparked an interest in using nutshells as an efficient, cost-effective sorbent for the removal of metals from contaminated waters.

The present study aims to expand the knowledge on the use of nutshells as efficient biosorbents for the remediation of cadmium, lead, and mercury from contaminated waters. Although previous studies such as [2,16,29,30,31,32] have already used nutshells to remove Cd and Pb from contaminated waters, the tested concentrations were unrealistically high based on the limits imposed by legislation, and concentrations found in the environment, which prevents a correct assessment of the viability of the biosorbent in a practical application [33]. Furthermore, the efficiency of nutshells to remove Hg from contaminated waters remains unstudied. As most studies also assess sorption efficiency solely in a distilled water matrix, the sorption behavior in a single- and multi-contaminated mineral water was also evaluated in the present study. The influence of a simple biosorbent pre-treatment (washing with 100 °C water) on the sorption efficiency was also addressed.

## 2. Materials and Methods

### 2.1. Reagents

All reagents were of pro-analytical quality obtained directly from suppliers, without any further modification. Standard stock solutions of the metals were purchased from Merck, namely nitrates of mercury (1001 ± 2 mg L^−1^), cadmium (1000 ± 2 mg L^−1^), and lead (1000 ± 2 mg L^−1^). Working solutions were obtained by diluting the corresponding stock solution. All the material used in the experiments was previously subjected to washing in Derquim 5%, rinsing in Milli-Q water (18 MΩ cm^−1^), soaking in HNO_3_ (25% *v*/*v*) for at least 24 h and subsequently rinsing with Milli-Q water. The glass vessels used in the experiments to store the water samples were additionally soaked in concentrated HNO_3_ (65% *v*/*v*) for at least 24 h before reuse.

### 2.2. Biosorbents Preparation

**Figure 1:**
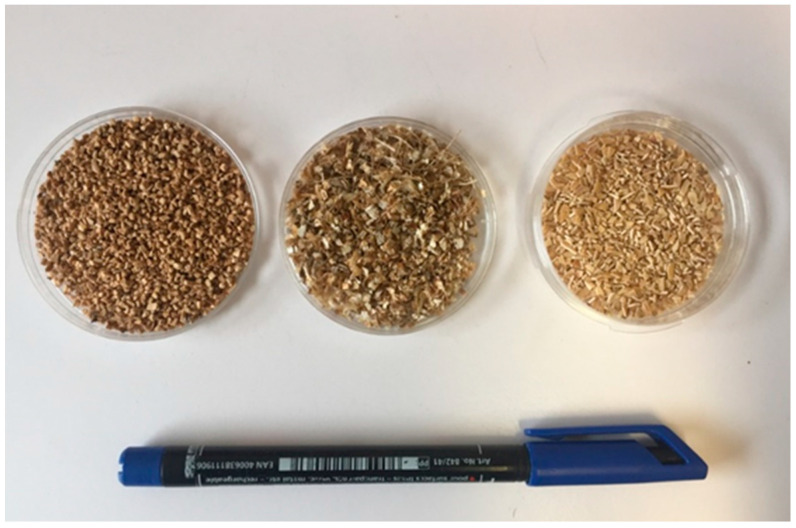
Sieved fraction (between 1 and 2 mm) of biosorbents (left to right: hazelnut, pistachio, walnut).

Almond, hazelnut, peanut, pistachio, and walnut shells used in the experiments were mechanically ground and sieved to obtain particles with sizes between 1 and 2 mm (Figure 1). Then, part of the material was washed abundantly with tap water at room temperature (22 °C), and another portion was washed with water at a temperature of 100 °C. These two washing procedures were considered as different biosorbent pre-treatments. After washing, materials were dried in a muffle at 35 °C for 48 h.

To better assess variations within elements and biosorbents, differences (Δ) between the *C_t_*/*C*_0_ ratios obtained for the biosorbents washed at 100 °C (R100) and 22 °C (R22) were calculated by the expression:

Δ = R100 − R22(1)

### 2.3. Nutshell Characterization by Fourier-Transform Infrared Spectroscopy

To identify the major functional groups responsible for the main sorption mechanisms, Fourier Transform Infrared Spectroscopy was performed on nutshells (previously washed at 100 °C), before and after being exposed to mono-element solutions containing 2 µmol L^−1^ of Cd, Pb, and Hg. Spectra were obtained on a Perkin Elmer Spectrum BX spectrometer coupled with a horizontal attenuated total reflectance cell (ATR). Readings were performed using 256 scans with a resolution of 4 cm^−1^ and spectra were represented as transmittance from 4000 to 600 cm^−1^.

### 2.4. Design of the Sorption Experiments

Biosorption experiments were performed in Schott Duran^®^ flasks of 1 L (reaction vessels), under magnetic stirring (450 rpm), at pH 6.5–7.0 (adjusted using a solution of NaOH 0.01 M), at room temperature (22 °C) for a maximum of 48 h. Equal amounts of each biosorbent (0.5 g) were added to individual spiked solutions of ultrapure water and mineral water. Cadmium (Cd), lead (Pb), and mercury (Hg) were chosen due to their potential toxicity (they are known as the toxic-trio [34,35]) and hence the need to be removed from waste water to protect the aquatic systems.

The efficiency of all biosorbents was tested in ultrapure water, where two spiked conditions were tested: (1) maximum concentrations allowed in wastewater discharges in Portugal (Decree-law 236/98 [10]), meaning mono-elemental spiked solution of 200 μg L^−1^ of Cd, 1000 μg L^−1^ of Pb, and 50 μg L^−1^ of Hg; (2) equal molar concentrations (2 μmol L^−1^) of Cd, Pb, and Hg corresponding to 225, 415, 400 μg L^−1^, respectively.

Under realistic wastewater conditions, other ions will be present along with Cd, Pb, and Hg (increased matrix complexity). These ions can influence the efficiency of the biosorbents by competing for the available sorption sites, which cannot be verified in ultrapure water. To assess the viability of the sorption process in more complex matrixes, experiments using natural mineral water spiked with Cd, Pb, and Hg were conducted for the most efficient biosorbent (hazelnut shells washed at 100 °C). Equimolar concentrations of 2 µmol L^−1^ of each element in mono and multi-contaminated systems were addressed (Table 1).

Controls, i.e., spiked solutions in absence of biosorbent were always run in parallel with the experiments with nutshells to evaluate possible loss of the studied elements in solution, due to independent factors, such as precipitation, adsorption to vessels walls, or volatilization [34]. Spiked solutions were left to pre-equilibrate for 12 h before the addition of biosorbents. Aliquots of 5 mL were collected at the following times: 0 (immediately before the addition of the biosorbents), 15 min, 30 min, 1, 3, 6, 9, 24, 48 h. After sampling, 25 μL of HNO_3_ (65% *v*/*v*) were added to obtain a pH < 2. Acidified samples were stored at 4 °C until the analysis.

### 2.5. Quantification of Cadmium, Lead, and Mercury in Solution

Cadmium and lead in solutions were quantified by inductively coupled plasma emission spectrometry (ICP-OES) on a Jobin Yvon Activa M. Calibration curves were obtained from 9 standards (0.1–100 μg L^−1^) prepared by diluting certified standard solutions of Cd(NO_3_)_2_ and Pb(NO_3_)_2_ in a solution of nitric acid (2% *v*/*v*). Calibration curves with correlation coefficients superior to 0.999 were considered, and standard coefficients of variation were always below 10%. Limits of quantification were 4 and 20 μg L^−1^ for Cd and Pb, respectively. Differences among replicates were below 10%. Cd and Pb in spiked water samples were measured directly or after dilution with HNO_3_ 2% [36].

The amount of mercury in solution was quantified by cold vapor atomic fluorescence spectroscopy (CV-AFS), on a PSA 10.025 Millennium Merlin Hg analyzer and using SnCl_2_ (2% m/v in HCl 10% *v*/*v*) as reducing agent. Calibration curves used five standard solutions of concentrations between 0.0 and 0.5 μg L^−1^. Standard solutions were prepared by diluting the stock solution of Hg 1000 mg L^−1^ in nitric acid (2% *v*/*v*). The limit of quantification was 0.1 μg L^−1^. Hg in spiked water samples was measured directly or after dilution with HNO_3_ 2% [34].

The quantity of Cd, Pb and Hg sorbed per unit of mass on the tested biosorbents (*q*, µg g^−1^) was estimated by the expression:

q = (C_0_ − C_t_) V/M, (2)

where C_0_ is the element concentration (µg L^−1^) at the initial time, C_t_ is the element concentration (µg L^−1^) in equilibrium, V is the volume of the solution (L), and M (g) is the mass of biosorbents.

### 2.6. Kinetic Modelling Applied to the Results

Two kinetics models, in their non-linear forms, pseudo-first order (PFO) model (3) and pseudo-second order (PSO) model (4) were adjusted to the experimental data obtained from the assays conducted with hazelnut shells. Both models have been widely applied in metal sorption studies using biological sorbents [34,37]. The PFO model is commonly associated with physisorption processes, while the PSO model suggests sorption of a chemical nature. To allow comparison among conditions, only the assays with element concentration of 2 µmol L^−1^ were analyzed.

(3) $$q_{t}=q_{e}\left(1-e^{-k1_{t}}\right)$$

(4) $$q_{t}=\frac{q_{e}^{2}k_{2}t}{1+q_{e}k_{2}t}$$

where q_e_ is the concentration of element on the sorbent at equilibrium (µg g^−1^), k_1_ (h^−1^) is the rate constant of pseudo-first order model, and k_2_ (g µg^−1^ h^−1^) is the rate constant of the pseudo-second order model. Detailed information of calculations can be found in [34,38].

## 3. Results

To minimize possible differences occurring among the spiked solutions at time zero, the concentration of each element in solution at time t (C_t_) was divided by the measured initial value (C_0_). Normalized concentrations in the present study correspond hence to the ratio C_t_/C_0_. Along the 48 h of contact, the concentrations of the controls varied less than 5%. The same variation was found among replicates.

### 3.1. Best Nutshells to Remove Cd, Pb, and Hg from Spiked Ultrapure Water

**Figure 2:**
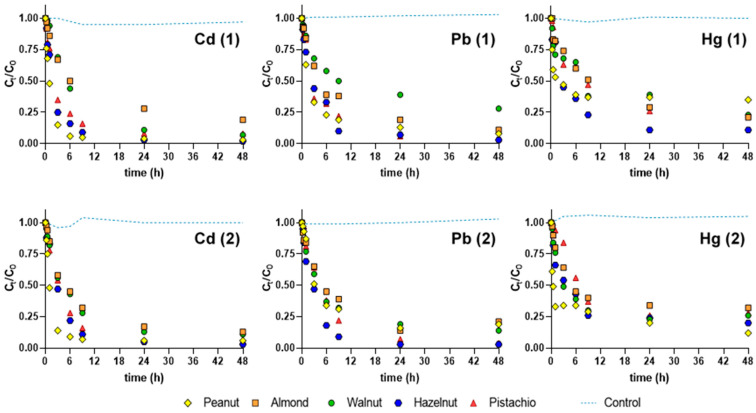
Normalized concentration (C_t_/C_0_) of cadmium (Cd), lead (Pb), and mercury (Hg) in ultrapure water spiked with initial concentrations of (1) 2 µmol L^−1^ and (2) 200 μg L^−1^ of Cd, 1000 μg L^−1^ of Pb, and 50 μg L^−1^ of Hg. Standard deviation bars (always ≤5%) were omitted for clarity.

Figure 2 shows the variation with time of the normalized concentrations (C_t_/C_0_) of Cd, Pb, and Hg in individual experiments with almonds, hazelnut, peanut, pistachio, and walnut shells pre-washed at 100 °C. Ultrapure water mono-element spiked with 50 μg L^−1^ of Hg, 200 μg L^−1^ of Cd and 1000 μg L^−1^ of Pb (maximum element concentrations for effluent discharges allowed in Portugal), as well as 2 µmol of Hg, Cd, and Pb (corresponding to 400, 225, 415 µg L^−1^, respectively) was used to assess the best biosorbents. All the biosorbents showed a fast removal of the elements in the first hours of contact, and after 9 h of exposure, approximately 70–75% of Cd, Pb, and Hg in the ultrapure water were removed. The decrease was slower between 24 and 48 h of contact. Experiments with almond and walnut shells showed approximately the same decline of Cd, Pb, and Hg normalized concentrations with time, which points to the similar removal rate of these elements by the two biosorbents. Peanuts, pistachio, and hazelnut removed less Hg than Cd or Pb.

Considering the removal rate at the beginning (up to 9 h), and the residual concentration in solution (C_t_/C_0_ at 48 h) it can be said that, overall, hazelnut was the fastest and most efficient among the tested biosorbents—Figure 2. After 9 h, hazelnut shells removed approximately 90% of Cd and Pb, and 75% of Hg, regardless of the initial concentration.

Table 2 gives the values of q for Cd, Pb, and Hg calculated for each biosorbent under the experimental conditions of V = 1 L and M = 0.5 g.

All biosorbents showed the ability to retain high quantities of Cd (q between 327 and 433 µg g^−1^), Pb (q between 1450 and 1725 µg g^−1^) and Hg (q between 506 and 680 µg g^−1^), mirroring the high initial concentration of 225, 1000, and 400 µg L^−1^ of each respective element in the experiment. Lower initial concentration was reflected in lower values of q. Removal percentages of Cd and Pb surpassed 80% for all biosorbents (except walnut for Pb). Hg removal efficiencies were generally lower, between 80% and 60% (except hazelnut, 90%). After 48 h of exposure to the spiking solutions, hazelnut showed the overall highest removal of Cd (98%) and Pb (97%), as well as for the highest concentration of Hg (90%).

### 3.2. Effect of Solution Chemistry on Removal of Cd, Hg, and Pb by Hazelnuts

**Figure 3:**
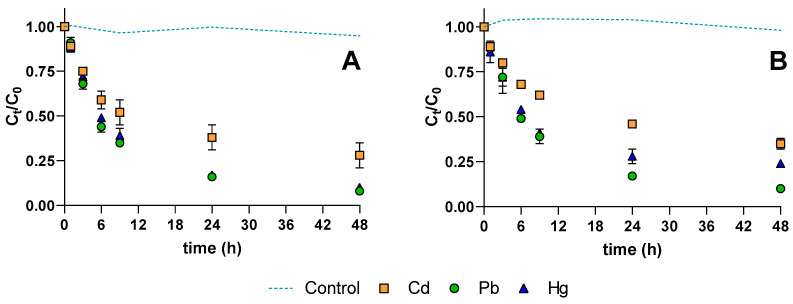
Normalized concentration (C_t_/C_0_) of cadmium (Cd), lead (Pb) and mercury (Hg) in mineral water spiked with 2 µmol L^−1^ of each element: (**A**) mono-element solution; (**B**) multi-element solution.

Due to the high sorption performance of hazelnut, this biosorbent was selected for assays with real mineral water, spiked with mono- or multi-elements (mixture of Cd, Hg, and Pb). The use of mineral water resulted from the need to understand the sorption process on more complex matrixes than “ideal” ultrapure water, to assess the viability of the proposed technology under realistic conditions. Figure 3A shows the variation with time of *C_t_*/*C*_0_ in mono-element spiked solutions of 2 μmol L^−1^ of Cd, Pb, and Hg. Results displayed (including control) correspond to the mean and standard deviation obtained from two replicates. Equal molar concentrations allow a comprehensive comparison of the sorption of Cd, Pb, and Hg by hazelnut shells.

After 48 h of contact, hazelnut removed 92% of Pb, 90% of Hg, and 73% of Cd. The percentages in mineral waters were in general lower than in ultrapure water. In mineral water solution containing simultaneously 2 μmol L^−1^ of Cd, Pb, and Hg (Figure 3B), removal after the 48 h reached 90% of Pb, 76% of Hg, and 65% of Cd, which are comparable to the ones observed in mono-spiked waters.

Mathematical modelling of the results obtained for hazelnut shells was performed. The goodness of the fits (evaluated by the coefficients of determination, R^2^) and calculated values of q_e_ are presented in Table 3.

The two models showed good coefficients of determination for most conditions (above 0.990), although worse adjustments were obtained for experiments conducted in ultrapure water. Thus, the modelling of sorption was not impaired by an increase in matrix complexity or by competition among Cd, Pb, and Hg. Higher values of qe were predicted by the PSO model. Although further studies are necessary to document the type of biosorption, the presented results are indicative in that both physisorption and chemisorption mechanisms may occur simultaneously [39].

### 3.3. Effect of Washing Procedure

Decreasing ratios C_t_/C_0_ along the experiments indicate adsorption onto the biosorbents. However, decreases differed with the temperature of the pre-treatment. Values of Δ (difference between normalized concentration with 100 °C and 22 °C) obtained for each element and biosorbent at t = 48 h are presented in Table 4. Differences below 10% were considered not relevant (n.r.).

Overall, small differences in removal performances were verified between nutshell pre-treatments. However, positive values of Δ in some conditions reveal a tendency for higher removal by nutshells washed at 100 °C. Hazelnut and walnut shells revealed higher Pb and Hg removal performance when washed at 100 °C, while almond and peanut shells displayed higher removals only for Hg. Pistachio shells did not display improved or impaired removal between pre-treatments.

Differences in the equimolar concentration suggest that rising temperature increased the sorption sites availability for Hg on the biosorbents (except for pistachio shells). No relevant differences were found in any biosorbents in Cd removal.

### 3.4. Major Functional Groups in Nutshells before and after Exposure to Cd, Pb, and Hg

**Figure 4:**
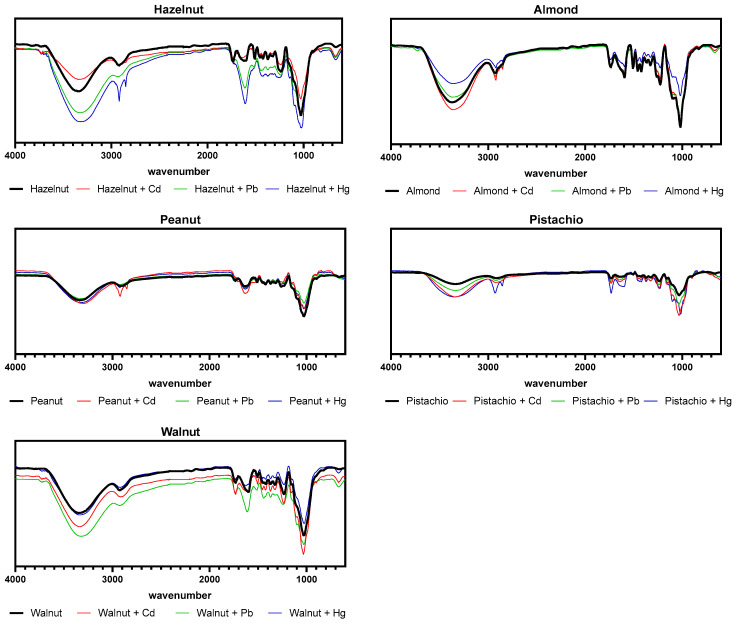
FTIR spectra of the biosorbents before and after exposure to mono-element (cadmium (Cd), lead (Pb) or mercury (Hg) spiked ultrapure water.

FTIR spectroscopy was used to identify the main functional groups present on the five biosorbents’ surfaces (Figure 4). All biosorbents showed common peaks associated with the main components present in most nutshells: cellulose, hemicellulose, and lignin.

The long band between 3000 and 3500 cm^−1^ corresponds to O–H stretching present in lignin, cellulose, and hemicellulose. The bands between 1025 and 1035 cm^−1^ and at around 1370 are associated with C–O stretching vibration of alcohols, carboxylic acids, and esters and C–H bending of alkenes, respectively, typically found in cellulose and hemicellulose [26,40,41]. Stretching vibrations of C=O bonds present in lignin and hemicellulose can be seen in the peaks at around 1735 cm^−1^ [41]. Characteristic peaks of lignin structure can be seen at 1605 cm^−1^, which corresponds to C=C aromatic groups [40]. All biosorbents also showed a peak near 1735 cm^−1^, which could be attributed to vibrations in acetyl or ester groups present in hemicellulose [26]. Additionally, all biosorbents (apart from walnut shells), verified an increase in sharpness of the peaks near 2916 and 2849 cm^−1^ after exposure, which can be attributed to CH and CH_2_ stretching of methyl and methylene groups [29].

Overall, all biosorbents revealed similar spectra, which qualitatively reflect their similar composition in terms of the main functional groups present. After exposure, changes in peak intensity were verified in the peaks at around 2916, 2849, and 1605 cm^−1^ for hazelnut shells, and to a lesser extent in the remaining biosorbents.

## 4. Discussion

### 4.1. Removal Differences among Elements and Biosorbents

Efficient removal of the toxic-trio was achieved by all biosorbents. However, when comparing element removal under equimolar conditions of 2 µmol L^−1^, maximum removal of Hg was lower than those of Cd or Pb. Furthermore, different removals were obtained among the five biosorbents, which suggests differences in biomass–metal affinities or in the mechanisms involved in the sorption process. It should be noted that although the studied nutshells share similar chemical composition in terms of the main functional groups identified, the relative proportion of the main components varies among them, which can influence sorption. While over 90% of Cd is removed by all biosorbents, except almond (81%), further differentiation can be observed for the remaining elements. Peanut (92%), pistachio (97%) and hazelnut (97%) was achieved for Pb removal, while for Hg only hazelnut shells reached 90% removal. These results reveal that hazelnut shells are the most viable biosorbent in the removal of all elements studied. Results agree with those obtained by [16] that achieved higher Pb removal with hazelnut (maximum of 28.18 mg g^−1^) than with almond shells (maximum of 8.08 mg g^−1^), pointing adsorption, ionic exchange, and chelation as the main sorption mechanisms. Although the identifiable peaks in the FTIR spectra of the present study revealed similarities, hazelnuts displayed the most significant changes after exposure to the elements, indicating the occurrence of chemisorption processes. It can be hypothesized that the superior efficiency of this biosorbent is related to the greater relevance that chemosorption has in the uptake compared to the other nutshells. Kinetic modelling of the results obtained for hazelnut shells in ultrapure water showed to be capable of accurately predicting the sorption process, nevertheless no information regarding the nature of the sorption mechanisms could be inferred. Also, biosorbent porous structure and mechanical resistance (not addressed in the present study) may affect removal. The higher efficiency of hazelnut shells could be due to its larger specific contact area in comparison to the other nutshells, which could only be related to a higher porosity, since the studies were carried out with the same amount of adsorbent, and with the same particle size. Data from the literature support this hypothesis as a higher Brunauer-Emmett-Teller (BET) surface area was reported for hazelnut shell (5.92 m^2^ g^−1^ [42]) than for peanut shell (2.005 m^2^ g^−1^ [43]), which were the best performing biosorbents in the present study.

Results obtained for the experiments ran in ultrapure water also revealed differences between the elements within the same sorbent (especially in the case of peanut and walnut shells). Although sorption is also influenced by solution parameters such as pH, ionic strength, and temperature, differences between metal affinity to the biosorbents may also be a combined result of individual metal characteristics such as ionic mobility, ionic radius, electronegativity, diffusion coefficient, and even hydration energy. In fact, ref. [2] attributed differences in the sorption of Pb and Cd onto modified peanut shells to such parameters. Also, ref. [44] used the hard-soft-acid-base (HSAB) concept to explain the removal sequence of Pb > Cd > Hg, that is, hard ”carboxyl base” in *Aspergillus niger* spores exhibit a higher affinity toward Pb (intermediate acid) than for Cd and Hg.

### 4.2. Effect of Ionic Strength and Metal Competition on the Removal of Cd, Pb, and Hg by Hazelnut Shells

A realistic application of any sorbent towards the remediation of a contaminated system will inevitably be conducted in the presence of complex matrixes where ionic strength, pH, and temperature may influence the sorbent’s efficiency. Ionic strength in particular can decrease the electrostatic forces between sorbent and sorbate due to competition among the cations present in solution [18,45]. This factor could explain the decrease observed in the present study on Cd removal in mineral water compared to ultrapure water, since the abundance of cations such as Ca^2+,^ Mg^2+^, and Na^+^ may inhibit the metal’s sorption. Furthermore, other elements such as Cl^−^ may have promoted the formation of stable complexes such as CdCl^+^, CdCl_2_, and CdCl_3_^−^, reducing the availability of Cd to be sorbed [46]. Pb and Hg were not affected by the increase in ionic strength, which may be a key property if hazelnut shells are used in the remediation of real contaminated waters. However, further increases in the ionic strength and pH variation should be studied to assess the versatility of this sorbent in a wide range of environmental conditions. Another factor that may influence the removal is the competition towards sorption sites by the contaminants themselves. It is essential to understand inter-contaminant interactions since multiple elements may be present in a contaminated effluent. In the present study, the transition from mono-contaminated to multi-contaminated water condition revealed a decrease in Hg removal by hazelnut shells by approximately 14%. Cd removal also decreased 7%, with a visible attenuation of the removal curve in the first 9 h of contact. Pb was the only element whose removal did not show any kind of impairment. The combined addition of Pb, Cd, and Hg to the mineral water resulted in an increase of ionic strength of 6 µmol L^−1^, which is negligible in comparison to the presence of other ions in solution. As such, it is plausible to suggest that the decrease in Hg and Cd sorption was the result of competition towards the sorbent’s binding sites. As previously mentioned, metal intrinsic characteristics can influence sorption behavior. As suggested by [47], the covalent index (CI, which is a function of electronegativity and ionic radius) can be a proxy for the potential of a given metal to establish covalent bonds with certain functional groups, in which the higher the covalent index, the higher that potential is. This notion is valid for the results obtained for Pb (CI_Pb_ = 3.287) and Cd (CI_Cd_ = 2.713), since not only was the removal for Pb higher, but its presence hindered the sorption of Cd. However, while Hg has an even higher covalent index (CI_Hg_ = 4.080), its removal was negatively influenced by the presence of Pb and Cd. This behavior can be further related to [48] classification of metals based on trends in the magnitude of equilibrium constants describing the formation of metal-ion complexes. While Hg^2+^ is classified as a class B metal, with a preferred sequence of ligands of S > N > O, Pb^2+^ and Cd^2+^ are classified as borderline metals, which can form complexes with a large array of ligands [48]. Hazelnut shells are composed mainly of cellulose, hemicellulose, and lignin without sulfur groups in the sorbent’s surface. The lack of preferential covalent ligands for Hg may thus counteract its higher covalent index in comparison to Pb, justifying lower removal. However, the covalent index is only indicative of the importance of covalent bonding relative to ionic interactions. As such, ionic interactions (i.e., electrostatic interactions) may still be an important source of sorption that cannot be disregarded. In fact, kinetic modelling of the results showed exceptional fits for both models generally associated with physisorption or chemisorption, corroborating the hypothesis of multiple sorption mechanisms acting simultaneously.

### 4.3. Effect of Pre-Treatment with Hot Water on Removal Efficiency of Biosorbents

Apart from pistachio shells, removal efficiency of the biosorbents was positively affected by washing with water at a temperature of 100 °C, with acccentuated differences for Hg. Changes in metal affinity are presumably related to the chemical structure of the biosorbents. FTIR results showed that the tested nutshells are mainly composed of lignin, cellulose, and hemicellulose. Although only qualitative results were obtained, the identification of the peaks is in line with the literature (Table 5), that indicates variations in the relative amounts of each component among nutshells. Previous studies have applied treatment of lignocellulosic biomass through acid, alkali, and hot water for several purposes. Acid treatment is efficient in hydrolyzing hemicellulose, breaking chemical bonds, and exposing cellulose, while alkali treatment efficiently removes lignin, effectively promoting degradation of the materials. In contrast, although the hot water treatment applied in the present study is simpler and less expensive, it is not effective in removing lignin from the lignocellulosic materials. Despite this, it can still remove some hemicellulose without promoting corrosion of the materials [49]. Washing at 100°C could also bring additional advantages, such as the enhancement on the release of phenolic compounds during washing that could interfere with the sorption process, as shown in [29].

Examples of the distinctive effects of pre-treatment of lignocellulosic biosorbents can be observed in [50] and in [51], where pre-treatment of walnut shells with citric acid increased the removal of Cr and Zn by four-fold and two-fold respectively, whereas pre-treatment with hot water in the present study only increased the removal efficiency of walnut for Pb and Hg between 11% and 24%. Pistachio shells did not display significant changes in removal efficiencies, which may be related to its highest cellulose and lowest lignin percentages among the five biosorbents. As it stands, although the pre-treatment with hot water may be a cheaper, cleaner method to increase metal removal by nutshells in comparison to the use of diluted acids, results indicate that a case-by-case approach is needed to guarantee the viability of the treatment in future applications.

### 4.4. Food and Agricultural Wastes as Biosorbents in the Remediation of Contaminated Waters

The use of food and agricultural wastes for the remediation of contaminated waters represents a growing interest in the scientific community as it belongs to the paradigm of treating waste with waste. As such, several studies have already shown the high efficiency of this type of waste in the removal of metal contaminants from waters. Besides nutshells, a variety of peels, shells, and husks have been used as sorbents to remove Cd and Pb. That is the case for banana peels [52], citrus peels [53], sweet potato peels [54], mangosteen shells [55], and plantain peels [56]. Removal of Hg is limited to a study using banana peels [37]. The main issues in the existing studies using food and agricultural wastes for the removal of Cd, Pb, and Hg derive from the unrealistic concentrations studied as well as the single-contaminant nature of the studies. In the present work, “environmentally relevant concentrations” are considered, which are in the same order of magnitude as the limits imposed by Portuguese Law on industrial effluent discharges. Apart from ref. [40] that focused on an environmentally relevant concentration of Hg (50 µg L^−1^), the remaining studies explore concentrations ranging from 15 to 500 mg L^−1^ of Cd and from 20 to 500 mg L^−1^ of Pb. The present study highlights the efficient removal of Cd, Pb, and Hg, in environmentally relevant concentrations, from contaminated waters by 0.5 g L^−1^ of hazelnut, walnut, almond, peanut, and pistachio shells. As for studies using nutshells in particular, hazelnut [16,30], almond [16], walnut [29,31], and peanut [2,32] shells have been used to remove Cd and Pb from contaminated waters (Table 6), while the removal efficiency of pistachio has so far only been assessed for chromium (Cr) [57]. To the best of our knowledge, the present study is the first to assess the viability of nutshells to remove Hg from contaminated waters.

In comparison, the kinetics of the present study is in general slower than that of previous studies. However, it should be noted that the higher the initial concentration of contaminant in solution results in a pronounced concentration gradient between the solution and the biosorbent (initially free of contaminant), leading to a greater driving force that raises the initial rate of biosorption [34]. Another important aspect to be considered is the residual concentration achieved, which in most cases remains excessively high for human consumption or reuse for irrigation, despite the high removal rates reported. Ref. [16] used 6 g L^−1^ of almond and hazelnut shells pre-treated at 100 ºC to remove 207 mg L^−1^ Pb from spiked distilled water. Authors found that only 4 h was necessary to remove 68% for almond and 90% for hazelnut. Yet, residual concentrations remained prohibitively high (in the order of mg/L). The current study shows that, using 12 times less biosorbent, more than 90% of Pb is removed from a solution, in 48 h, leading to levels of this metal in solution that are in accordance with the legal criterion for water for human consumption in Portugal (10 µg L^−1^, [11]).

Another differentiating aspect between the present study and previous studies is the chosen mass of biosorbent. Only 0.5 g L^−1^ of biosorbent were used here, as opposed to the 1–15 g L^−1^ observed for other studies. Despite the lower mass of biosorbent, results showed that it was possible to remove over 90% of each element from initial concentrations deemed as environmentally relevant. As such, by reducing biosorbent dosage without compromising the removal process, smaller amounts of sludge would be produced in an up-scale experiment. Although reusability of the nutshells was not assessed, the abundance and inexpensive nature of these wastes makes them almost inexhaustible in a future application, as compared to other materials such as synthetic nanomaterials [58] or resins [59,60]. Even so, the possibility of sorbent reuse through contaminant desorption should not be excluded, as it has already been shown to be possible for lignocellulosic biomass [61,62,63].

## 5. Conclusions

This study indicates a simple and efficient procedure to improve the quality of wastewater by removing Cd, Pb, and Hg using nutshells. Compared to previous studies, a smaller amount of biosorbents was used which may contribute to a better management of waste by-products. Also, unlike most previous studies, environmentally relevant concentrations were considered. After 48 h, the concentration of these elements in the treated water is compatible to its use for other purposes contributing to the water reuse. Further studies with real wastewater will help to define tailored solutions for various case studies. Although all biosorbents showed highly efficient removal of Pb, Cd, and Hg, hazelnut shells stood out as the overall best biosorbent. The effects of biomass pre-treatment, consisting of a boiling water washing, revealed the process to be environmentally and economically viable in increasing contaminant removal. By increasing matrix complexity, factors such as ionic strength and complexation with other ions present in solution resulted in a decrease of Cd removal, although Pb and Hg were not affected. Lower electronegativity and ionic radius may also justify lower Cd removal. When present in the same solution in equimolar concentrations, Pb and Hg competed for binding sites on the biosorbent’s surface, resulting in Hg sorption impairment. Presumably, the preference towards Pb was a result of the high availability of sorption sites with exceptional affinity towards this borderline class metal. Overall, nutshells proved to be a promising solution to the remediation of Pb, Cd, and Hg contaminated water. Further work concerning the effects of increased ionic strength, varying pH, interactions with other contaminants, as well as a life-cycle assessment are proposed as methods to evaluate the possibility of nutshells acting as a global low-cost, environmentally friendly remediator for contaminated waters.

## Figures and Tables

**Table 1:** Experimental conditions of the conducted biosorption assays.

		Hazelnut	Almond	Peanut	Pistachio	Walnut
**Ultrapure water**	Mono-element	x	x	x	x	x
	Equimolar					
	Mono-element	x	x	x	x	x
	Discharge					
**Mineral water**	Mono-element	x				
	Equimolar					
	Mono-element	x				
	Discharge					

**Table 2:** Quantity of cadmium (Cd), lead (Pb), and mercury (Hg) sorbed per unit of mass onto the biosorbents surface (q, µg g^−1^) and respective percentage of removal (%) relative to the initial concentration.

		Cd		Pb		Hg	
		2 µmol L^−1^	200 µg L^−1^	2 µmol L^−1^	1000 µg L^−1^	2 µmol L^−1^	50 µg L^−1^
		(225 µg L^−1^)		(415 µg L^−1^)		(400 µg L^−1^)	
Hazelnut	q (µg g^−1^)	425	324	714	1725	680	63
	Removal (%)	98	97	97	97	90	78
Almond	q (µg g^−1^)	327	354	679	1570	607	67
	Removal (%)	81	87	89	79	79	68
Peanut	q (µg g^−1^)	433	386	729	1414	506	89
	Removal (%)	97	94	92	81	65	88
Pistachio	q (µg g^−1^)	389	331	669	1450	633	62
	Removal (%)	88	94	91	96	77	72
Walnut	q (µg g^−1^)	378	373	480	1706	609	71
	Removal (%)	93	89	72	86	77	74

**Table 3:** Correlation coefficients (R^2^) and value of q at equilibrium (q_e_) retrieved from the pseudo-first (PFO) and pseudo-second (PSO) order kinetic models for each of the experiments with hazelnut shells and cadmium (Cd), lead (Pb) and mercury (Hg).

		PFO			PSO		
		R^2^	k_1_	q_e_ (µg g^−1^)	R^2^	k_2_	q_e_ (µg g^−1^)
**Cd**	Ultrapure	0.990	0.423	408	0.977	1.29 × 10^−3^	449
	Mineral	0.992	0.144	297	0.999	4.60 × 10^−4^	349
	Mineral Mix	0.986	0.115	265	0.997	3.82 × 10^−4^	319
**Pb**	Ultrapure	0.985	0.269	693	0.987	4.89 × 10^−4^	767
	Mineral	0.995	0.150	703	0.992	2.01 × 10^−4^	826
	Mineral Mix	0.998	0.135	670	0.996	1.84 × 10^−4^	795
**Hg**	Ultrapure	0.986	0.265	656	0.987	4.86 × 10^−4^	731
	Mineral	0.993	0.161	529	0.996	3.00 × 10^−4^	615
	Mineral Mix	0.996	0.170	571	0.993	3.08 × 10^−4^	656

**Table 4:** Differences (Δ) in removal efficiency based on pre-treatment (100 °C and 22 °C) for the different elements (cadmium (Cd), lead (Pb) and mercury (Hg) and biosorbents (Hazelnut, Almond, Peanut, Pistachio and Walnut), for the two contamination scenarios studied (Equimolar and Discharge).

		Hazelnut	Almond	Peanut	Pistachio	Walnut
**Equimolar**	Cd	n.r.	n.r.	n.r.	n.r.	n.r.
	Pb	0.10	n.r.	n.r.	n.r.	0.11
	Hg	0.16	0.19	0.16	n.r.	0.24
**Discharge**	Cd	n.r.	n.r.	n.r.	n.r.	n.r.
	Pb	0.13	n.r.	n.r.	n.r.	0.22
	Hg	0.21	n.r.	0.23	n.r.	n.r.

**Table 5:** Percentage of lignin, cellulose, and hemicellulose in the tested nutshells compiled from literature.

	Hazelnut ^a,b,c^	Almond ^d,e^	Peanut ^a,f^	Pistachio ^d,g^	Walnut ^d,h^
Lignin	29.6–40.1	25.5–39.3	27.6–30.6	16.3–23.6	27.2–43.7
Cellulose	22.9–37.5	34.4–38.5	37.5–38.9	37.5–38.1	36.4–42.4
Hemicellulose	12.7–24.9	14.0–28.8	15.4–16.3	25.3–31.4	10.3–27.9

^a^ [54]; ^b^ [55]; ^c^ [56]; ^d^ [30]; ^e^ [57]; ^f^ [58]; ^g^ [59].

**Table 6:** Main experimental conditions of studies using nutshells as biosorbents to remove Cd and Pb from contaminated water.

Reference	Biosorbent	Matrix	Contaminant	Maximum Removal
[30]	Hazelnut (4 g L^−1^)	Distilled water	Cd 14.5 mg L^−1^	92.4% (5 h)
[16]	Hazelnut (6.3 g L^−1^) Almond (6.3 g L^−1^)	Distilled water	Pb 207 mg L^−1^	Hazelnut: 90% (4 h) Almond: 68% (4 h)
[31]	Walnut (2 g L^−1^)	Distilled water	Cd 100 mg L^−1^	45% (3.3 h)
[29]	Pecan nut (1–15 g L^−1^)	Distilled water	Pb 100 mg L^−1^	95% (6 h)
[32]	Peanut (5 g L^−1^)	Wastewater	Cd 46 mg L^−1^ Pb 0.26 mg L^−1^	Cd: 38% (1 h) Pb: <99% (1 h)
[2]	Peanut (4 g L^−1^)	Deionized water	Cd 500 mg L^−1^ Pb 500 mg L^−1^	Cd: 6.14% (1 h) Pb: 22.6% (1 h)

## References

[1] Febrianto J., Kosasih A.N., Sunarso J., Ju Y.H., Indraswati N., Ismadji S. (2009). Equilibrium and kinetic studies in adsorption of heavy metals using biosorbent: A summary of recent studies. J. Hazard. Mater..

[2] Rozumová L., Životský O., Seidlerová J., Motyka O., Šafařík I., Šafaříková M. (2016). Magnetically modified peanut husks as an effective sorbent of heavy metals. J. Environ. Chem. Eng..

[3] Adamu C.I., Nganje T.N., Edet A. (2015). Heavy metal contamination and health risk assessment associated with abandoned barite mines in Cross River State, southeastern Nigeria. Environ. Nanotechnol. Monit. Manag..

[4] Chowdhury S., Mazumder M.A.J., Al-Attas O., Husain T. (2016). Heavy metals in drinking water: Occurrences, implications, and future needs in developing countries. Sci. Total Environ..

[5] Jiang L., Zhou W., Liu D., Liu T., Wang Z. (2017). Biosorption isotherm study of Cd2+, Pb2+ and Zn2+ biosorption onto marine bacterium Pseudoalteromonas sp. SCSE709-6 in multiple systems. J. Mol. Liq..

[6] Farooq U., Kozinski J.A., Khan M.A., Athar M. (2010). Biosorption of heavy metal ions using wheat based biosorbents—A review of the recent literature. Bioresour. Technol..

[7] Abdullah E.J. (2013). Quality Assessment for Shatt Al-Arab River Using Heavy Metal Pollution Index and Metal Index. J. Environ. Earth Sci..

[8] Singh U.K., Kumar B. (2017). Pathways of heavy metals contamination and associated human health risk in Ajay River basin, India. Chemosphere.

[9] WHO (2017). Guidelines for Drinking-Water Quality.

[10] (1998). Decree-Law No. 236/98 of the Portuguese Ministry of the Environment of 1 August Establishing Water Quality Standards. Diário da República I Série.

[11] (2017). AMBIENTE Decree-Law No. 152/2017. Diário da República.

[12] Agency for Toxic Substances and Disease Registry ATSDR’s Substance Priority List. https://www.atsdr.cdc.gov/spl/#2019spl.

[13] Witek-Krowiak A., Szafran R.G., Modelski S. (2011). Biosorption of heavy metals from aqueous solutions onto peanut shell as a low-cost biosorbent. Desalination.

[14] Peng S.H., Wang R., Yang L.Z., He L., He X., Liu X. (2018). Biosorption of copper, zinc, cadmium and chromium ions from aqueous solution by natural foxtail millet shell. Ecotoxicol. Environ. Saf..

[15] Yahaya Y.A., Mat Don M., Bhatia S. (2009). Biosorption of copper (II) onto immobilized cells of Pycnoporus sanguineus from aqueous solution: Equilibrium and kinetic studies. J. Hazard. Mater..

[16] Pehlivan E., Altun T., Cetin S., Iqbal Bhanger M. (2009). Lead sorption by waste biomass of hazelnut and almond shell. J. Hazard. Mater..

[17] Wang J.L., Chen C. (2009). Biosorbents for heavy metals removal and their future. Biotechnol. Adv..

[18] Fomina M., Gadd G.M. (2014). Biosorption: Current perspectives on concept, definition and application. Bioresour. Technol..

[19] Robalds A., Naja G.M., Klavins M. (2016). Highlighting inconsistencies regarding metal biosorption. J. Hazard. Mater..

[20] Ismail I., Moustafa T. (2016). Biosorption of heavy metals. Heavy Metals: Sources, Toxicity and Remediation Techniques.

[21] Gadd G.M. (2009). Biosorption: Critical review of scientific rationale, environmental importance and significance for pollution treatment. J. Chem. Technol. Biotechnol..

[22] Dai Y., Sun Q., Wang W., Lu L., Liu M., Li J., Yang S., Sun Y., Zhang K., Xu J. (2018). Utilizations of agricultural waste as adsorbent for the removal of contaminants: A review. Chemosphere.

[23] De Freitas G.R., da Silva M.G.C., Vieira M.G.A. (2019). Biosorption technology for removal of toxic metals: A review of commercial biosorbents and patents. Environ. Sci. Pollut. Res..

[24] Pavan F.A., Lima I.S., Lima É.C., Airoldi C., Gushikem Y. (2006). Use of Ponkan mandarin peels as biosorbent for toxic metals uptake from aqueous solutions. J. Hazard. Mater..

[25] Sud D., Mahajan G., Kaur M.P. (2008). Agricultural waste material as potential adsorbent for sequestering heavy metal ions from aqueous solutions—A review. Bioresour. Technol..

[26] Maaloul N., Oulego P., Rendueles M., Ghorbal A., Díaz M. (2017). Novel biosorbents from almond shells: Characterization and adsorption properties modeling for Cu (II) ions from aqueous solutions. J. Environ. Chem. Eng..

[27] International Nut & Dried Fruit Council (2020). Nuts & Dried Fruits—Statistical Yearbook 2019–2020. Stat. Yearb..

[28] Khir R., Pan Z. (2019). Walnuts. Integrated Processing Technologies for Food and Agricultural By-Products.

[29] Vaghetti J.C.P., Lima E.C., Royer B., da Cunha B.M., Cardoso N.F., Brasil J.L., Dias S.L.P. (2009). Pecan nutshell as biosorbent to remove Cu (II), Mn (II) and Pb (II) from aqueous solutions. J. Hazard. Mater..

[30] Cimino G., Passerini A., Toscano G. (2000). Removal of toxic cations and Cr (VI) from aqueous solution by hazelnut shell. Water Res..

[31] Feizi M., Jalali M. (2015). Removal of heavy metals from aqueous solutions using sunflower, potato, canola and walnut shell residues. J. Taiwan Inst. Chem. Eng..

[32] Abdelfattah I., Ismail A.A., Sayed F.A., Almedolab A., Aboelghait K.M. (2016). Biosorption of heavy metals ions in real industrial wastewater using peanut husk as efficient and cost effective adsorbent. Environ. Nanotechnol. Monit. Manag..

[33] Bessa A., Henriques B., Gonçalves G., Irurueta G., Pereira E., Marques P.A.A.P. (2020). Graphene oxide/polyethyleneimine aerogel for high-performance mercury sorption from natural waters. Chem. Eng. J..

[34] Henriques B., Lopes C.B., Figueira P., Rocha L.S., Duarte A.C., Vale C., Pardal M.A., Pereira E. (2017). Bioaccumulation of Hg, Cd and Pb by Fucus vesiculosus in single and multi-metal contamination scenarios and its effect on growth rate. Chemosphere.

[35] Henriques B., Rodrigues S.M., Coelho C., Cruz N., Duarte A.C., Römkens P.F.A.M., Pereira E. (2013). Risks associated with the transfer of toxic organo-metallic mercury from soils into the terrestrial feed chain. Environ. Int..

[36] Costa M., Henriques B., Pinto J., Fabre E., Viana T., Ferreira N., Amaral J., Vale C., Pinheiro-Torres J., Pereira E. (2020). Influence of salinity and rare earth elements on simultaneous removal of Cd, Cr, Cu, Hg, Ni and Pb from contaminated waters by living macroalgae. Environ. Pollut..

[37] Fabre E., Lopes C.B., Vale C., Pereira E., Silva C.M. (2020). Valuation of banana peels as an effective biosorbent for mercury removal under low environmental concentrations. Sci. Total Environ..

[38] Ho Y.-S. (2006). Review of second-order models for adsorption systems. J. Hazard. Mater..

[39] Ho Y.S., Ng J.C.Y., Mckay G. (2000). Kinetics of pollutant sorption by biosorbents: Review. Sep. Purif. Methods.

[40] Gozaydin G., Yuksel A. (2017). Valorization of hazelnut shell waste in hot compressed water. Fuel Process. Technol..

[41] Li X., Liu Y., Hao J., Wang W. (2018). Study of almond shell characteristics. Materials.

[42] Şencan A., Karaboyacı M., Kılıç M. (2015). Determination of lead (II) sorption capacity of hazelnut shell and activated carbon obtained from hazelnut shell activated with ZnCl2. Environ. Sci. Pollut. Res..

[43] Li R., Zhang Y., Chu W., Chen Z., Wang J. (2018). Adsorptive removal of antibiotics from water using peanut shells from agricultural waste. RSC Adv..

[44] Cui H., Liu X., Li K., Cao T.T., Cui C., Wang J.Y. (2020). Mechanism of Hg (II), Cd (II) and Pb (II) ions sorption from aqueous solutions by Aspergillus niger spores. Sep. Sci. Technol..

[45] Costa M., Henriques B., Pinto J., Fabre E., Dias M., Soares J., Carvalho L., Vale C., Pinheiro-Torres J., Pereira E. (2020). Influence of toxic elements on the simultaneous uptake of rare earth elements from contaminated waters by estuarine macroalgae. Chemosphere.

[46] Powell K.J., Brown P.L., Byrne R.H., Gajda T., Hefter G., Leuz A.K., Sjöberg S., Wanner H. (2011). Chemical speciation of environmentally significant metals with inorganic ligands. Part 4: The Cd2+ + OH-, Cl-, co32-, So42-, and Po43- systems (IUPAC technical report). Pure Appl. Chem..

[47] Brady J.M., Tobin J.M. (1995). Binding of hard and soft metal ions to Rhizopus arrhizus biomass. Enzyme Microb. Technol..

[48] Nieboer E., Richardson D.H.S. (1980). The replacement of the nondescript term “heavy metals” by a biologically and chemically significant classification of metal ions. Environ. Pollution. Ser. B Chem. Phys..

[49] Zheng Q., Zhou T., Wang Y., Cao X., Wu S., Zhao M., Wang H., Xu M., Zheng B., Zheng J. (2018). Pretreatment of wheat straw leads to structural changes and improved enzymatic hydrolysis. Sci. Rep..

[50] Altun T., Pehlivan E. (2012). Removal of Cr (VI) from aqueous solutions by modified walnut shells. Food Chem..

[51] Segovia-Sandoval S.J., Ocampo-Pérez R., Berber-Mendoza M.S., Leyva-Ramos R., Jacobo-Azuara A., Medellín-Castillo N.A. (2018). Walnut shell treated with citric acid and its application as biosorbent in the removal of Zn (II). J. Water Process Eng..

[52] Anwar J., Shafique U., Waheed-uz-Zaman, Salman M., Dar A., Anwar S. (2010). Removal of Pb (II) and Cd (II) from water by adsorption on peels of banana. Bioresour. Technol..

[53] Schiewer S., Balaria A. (2009). Biosorption of Pb2+ by original and protonated citrus peels: Equilibrium, kinetics, and mechanism. Chem. Eng. J..

[54] Asuquo E.D., Martin A.D. (2016). Sorption of cadmium (II) ion from aqueous solution onto sweet potato (Ipomoea batatas L.) peel adsorbent: Characterisation, kinetic and isotherm studies. J. Environ. Chem. Eng..

[55] Zein R., Suhaili R., Earnestly F., Indrawati, Munaf E. (2010). Removal of Pb (II), Cd (II) and Co (II) from aqueous solution using Garcinia mangostana L. fruit shell. J. Hazard. Mater..

[56] Garba Z.N., Ugbaga N.I., Abdullahi A.K. (2016). Evaluation of optimum adsorption conditions for Ni (II) and Cd (II) removal from aqueous solution by modified plantain peels (MPP). Beni-Suef Univ. J. Basic Appl. Sci..

[57] Moussavi G., Barikbin B. (2010). Biosorption of chromium (VI) from industrial wastewater onto pistachio hull waste biomass. Chem. Eng. J..

[58] Wu Y., Pang H., Liu Y., Wang X., Yu S., Fu D., Chen J., Wang X. (2019). Environmental remediation of heavy metal ions by novel-nanomaterials: A review. Environ. Pollut..

[59] Freitas O.M.M., Martins R.J.E., Delerue-Matos C.M., Boaventura R.A.R. (2008). Removal of Cd (II), Zn (II) and Pb (II) from aqueous solutions by brown marine macro algae: Kinetic modelling. J. Hazard. Mater..

[60] Shaaban A.F., Fadel D.A., Mahmoud A.A., Elkomy M.A., Elbahy S.M. (2013). Synthesis and characterization of dithiocarbamate chelating resin and its adsorption performance toward Hg (II), Cd (II) and Pb (II) by batch and fixed-bed column methods. J. Environ. Chem. Eng..

[61] Rambabu K., Thanigaivelan A., Bharath G., Sivarajasekar N., Banat F., Show P.L. (2020). Biosorption potential of Phoenix dactylifera coir wastes for toxic hexavalent chromium sequestration. Chemosphere.

[62] Hossain M.A., Ngo H.H., Guo W.S., Setiadi T. (2012). Adsorption and desorption of copper (II) ions onto garden grass. Bioresour. Technol..

[63] Abdolali A., Guo W.S., Ngo H.H., Chen S.S., Nguyen N.C., Tung K.L. (2014). Typical lignocellulosic wastes and by-products for biosorption process in water and wastewater treatment: A critical review. Bioresour. Technol..

